# A Case Report on Cerebral Venous Sinus Thrombosis With Factor V Leiden Mutation in the Postpartum Period

**DOI:** 10.7759/cureus.80158

**Published:** 2025-03-06

**Authors:** Oluwasegun A Shoewu, Jevon Harrison, Auda H Auda, Juan Escobar, Monica Liriano, Iyad Baker

**Affiliations:** 1 Family Medicine, Hackensack University Medical Center, Hackensack, USA; 2 General Internal Medicine, Hackensack Meridian Health Palisades Medical Center, North Bergen, USA; 3 Internal Medicine, Hackensack Meridian Health Palisades Medical Center, North Bergen, USA; 4 Obstetrics and Gynaecology, Hackensack Meridian Health Palisades Medical Center, North Bergen, USA; 5 Family Medicine, Hackensack Meridian Health Palisades Medical Center, North Bergen, USA

**Keywords:** anticoagulation, cerebral venous sinus thrombosis (cvst), heterozygous factor v leiden mutation, migraine, postpartum headache

## Abstract

Cerebral venous sinus thrombosis (CVST) remains an elusive diagnosis in the pregnancy and postpartum periods requiring heightened clinical vigilance due to its potential for severe morbidity and mortality, including seizures, strokes, and death. This neurologic emergency typically responds well to anticoagulation therapy, though some cases may require endovascular interventions like thrombectomy. Here, we describe the case of a 28-year-old female patient who was two weeks postpartum at the time of presentation and had a history of migraines responsive to over-the-counter medication, presenting with persistent headaches unresponsive to typical analgesics which she typically uses for her migraines. She was diagnosed with CVST, which was further complicated by a heterozygous factor V Leiden mutation. The patient exhibited a favorable response to anticoagulation treatment.

## Introduction

Cerebral venous sinus thrombosis (CVST) is a rare neurovascular emergency caused by the formation of blood clots in the cerebral venous sinuses. Despite its rarity, with an estimated incidence reported as 0.0003%, CVST often goes undiagnosed due to its subtle and varied symptoms, making it a critical condition with potentially severe outcomes if missed [1]. The disorder predominantly affects the brain’s venous circulation, manifesting through symptoms that include headaches, which are the most common symptom, neck stiffness, visual disturbances like blurry vision or photophobia, and more severe outcomes such as fainting, nausea, vomiting, strokes, seizures, and in extreme cases, death. CVST is most frequently observed in women under the age of 50, and risk factors encompass a range of conditions including hypercoagulable states, pregnancy, puerperium, contraceptives, trauma, thrombophilia, previous venous thromboembolic episodes, malignancy, and neurosurgical interventions [2]. Notably, the factor V Leiden mutation or any other factor that increases the risk of coagulation will significantly increase the risk of thromboembolic events, emphasizing the need for diligent diagnosis, particularly when patients present with atypical symptoms or when symptoms persist despite standard treatment [3].

## Case presentation

A 28-year-old woman, two weeks postpartum, presented to the emergency department with elevated blood pressure and a four-day history of headaches, described as different from her typical migraines, which were generally relieved by Ibuprofen. Her typical headaches present as unilateral constant throbbing pain and are not more than 5 out of 10 in severity with no blurring vision or eye swelling. These new headaches were generalized, ranging in severity from moderate to severe, lasting more than 5 out of 10 in pain severity and were accompanied by occasional eye swelling, blurry vision, and palpitations. 

Medical background

Her medical history included migraines and a past infection with Herpes Simplex Virus (type 1) which had been successfully treated. No present infection with Herpes at this time. She reported no use of oral contraceptives or history of cardiac abnormalities, blood disorders, autoimmune diseases, or tumors. She had quit smoking two years prior and denied current alcohol or illicit drug use.

Clinical examination

Upon examination, her vitals were noted as follows: blood pressure 137/102 mmHg, pulse 76 bpm, temperature 98.5°F, respiration rate 18 breaths per minute, and oxygen saturation at 100%. Neurological assessment showed that she was oriented in time, place, and person and had no focal deficits.

Diagnostic evaluation

Laboratory tests done during the first few days in the hospital, including complete blood count, comprehensive metabolic panel, thyroid function, and coagulation panel, were normal. An electrocardiogram confirmed sinus rhythm without abnormalities. Doppler scans for deep vein thrombosis were negative. Echocardiography showed normal cardiac structure and function with no evidence of septal defects or significant valve disease. Genetic testing was done a few days after the resolution of symptoms and it revealed a heterozygous mutation in the factor V gene, indicating an increased risk for venous thrombosis.

Imaging findings

**Figure 1:**
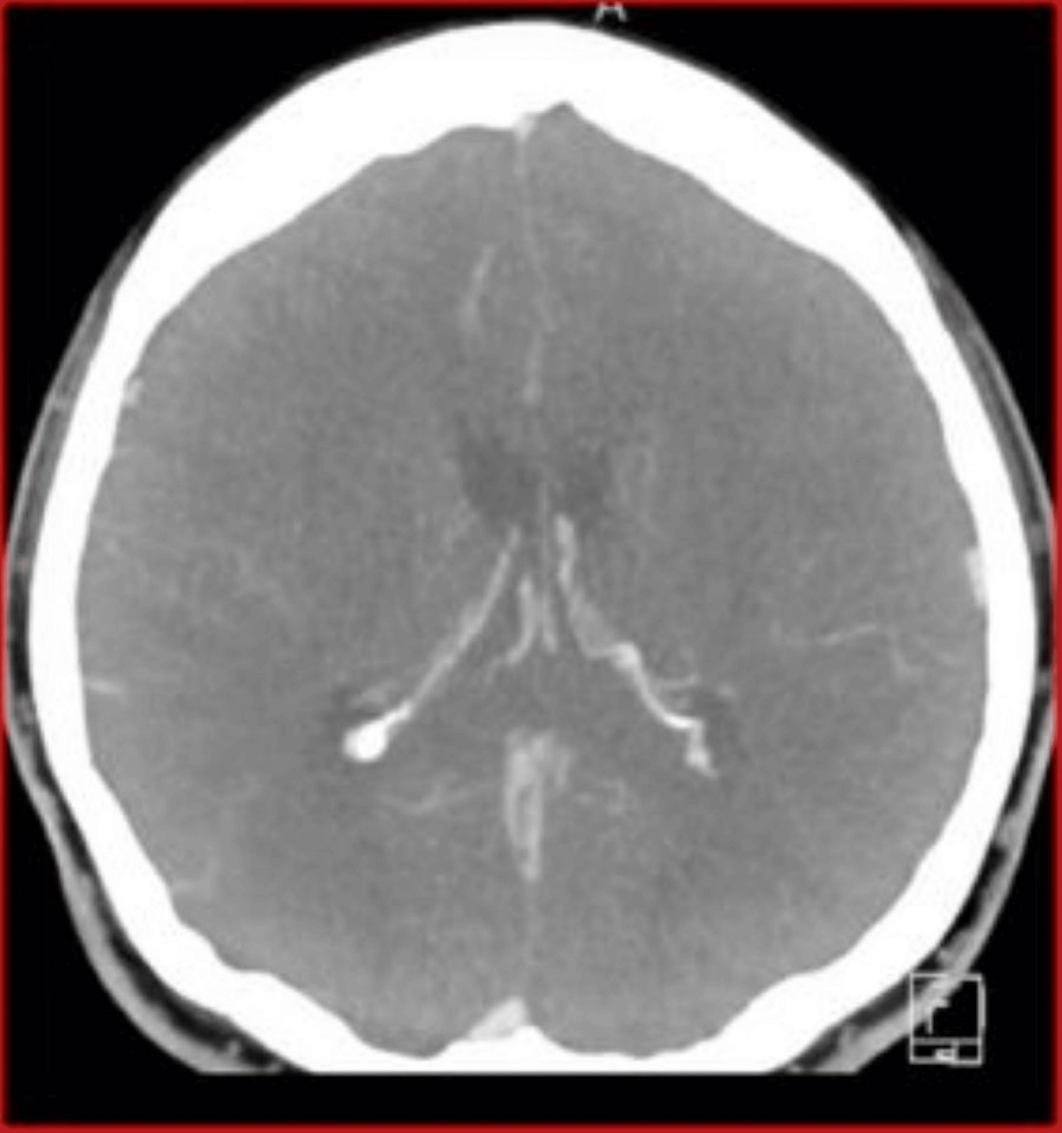
CT venogram with no blockages identified

**Figure 2:**
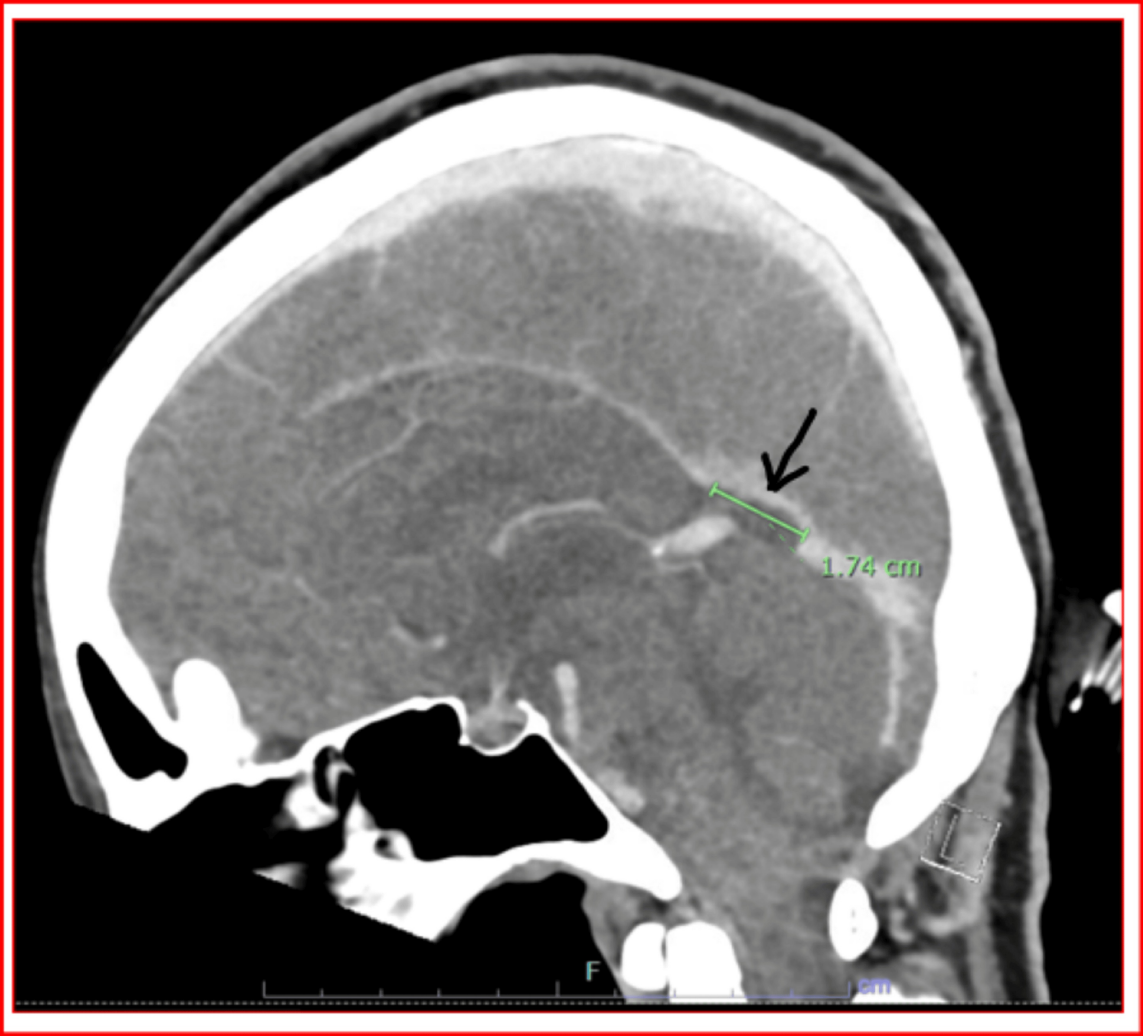
CT venogram showing focal filling defect up to 1.7 cm at the level of the proximal straight sinus, suggestive of thrombus formation and normal venous return in distal straight sinus with a visible right internal jugular vein

CT venography was performed, yielding several crucial insights. As shown in Figure 1, the superior sagittal sinus, internal cerebral veins, and vein of Galen are patent, indicating no blockages in these major venous channels. At the level of the proximal straight sinus, there is a notable focal filling defect measuring up to 1.7 cm, suggestive of thrombus formation (Figure 2). The distal straight sinus shows expected enhancement, indicating a normal venous return in that segment.

The imaging also reveals an asymmetry of the transverse sinuses, with the right sinus appearing more robust than the left a feature that may be developmental. Similar asymmetry is observed in the sigmoid sinuses, though they remain patent. A prominently visible right internal jugular vein is also noted (Figure 2).

Diagnosis and management

The radiological findings led to a diagnosis of CVST. The patient was admitted to the intensive care unit for close monitoring and started on anticoagulation therapy with heparin and later transitioned to subcutaneous low-molecular-weight heparin. The treatment with heparin will be continued for up to 90 days considering the fact that the patient was breast feeding at that time. Antihypertensives and medications for pain management were also administered. Her condition improved significantly within 24 hours, and she was scheduled for follow-up with her primary care provider and obstetrician. A repeat CT venogram done seven months after showed the resolution of CVST.

## Discussion

The case of a 28-year-old postpartum female patient presenting with CVST underscores the complex interplay of physiological changes during the postpartum period and genetic predispositions such as the factor V Leiden mutation. CVST, though rare with an incidence of about 0.0005%, poses significant diagnostic challenges due to its diverse and non-specific symptomatology, often mimicking less severe conditions like migraines [1,4].

In this case, the presence of the factor V Leiden mutation heightened the patient's risk for thromboembolic events, aligning with findings that suggest a gender-dependent predisposition to venous thrombosis in individuals with this mutation [3]. The management of CVST involves prompt anticoagulation to prevent further thrombosis and manage existing clots, as demonstrated in this case where early intervention led to a rapid improvement in symptoms [5].

Imaging plays a crucial role in diagnosing CVST, with CT venography providing detailed views of venous structures and potential anomalies. MRI (magnetic resonance imaging), which is a superior imaging technique, can also be utilized in detecting CVST. The detection of a thrombus in the cerebral veins, which is the proximal straight sinus in our patient, highlighted the need for aggressive management, consistent with guidelines suggesting the pivotal role of imaging in confirming the diagnosis of CVST [2,6].

The implications for future care involve not only ongoing management of the thrombotic condition but also genetic counseling for the patient and potentially affected family members, given the hereditary nature of factor V Leiden mutation. This case exemplifies the need for awareness and consideration of CVST in differential diagnoses, particularly in postpartum women presenting with atypical headaches and visual symptoms [7,8].

## Conclusions

This case of CVST in a postpartum female patient with factor V Leiden mutation illustrates the critical importance of considering CVST in differential diagnoses for postpartum women presenting with unusual headaches. It underscores the necessity of timely and appropriate imaging and genetic testing to guide effective management strategies. This case highlights the importance of multidisciplinary care involving obstetrics, neurology, and hematology to optimize patient outcomes. Moreover, it serves as a reminder of the potential genetic implications of thrombophilia, advocating for family screening and genetic counseling where relevant. The limited available resources prevented evaluation of more genetic prothrombotic components and uncommon hematological disorders. Future research could further explore preventive strategies and the role of genetic predispositions in CVST to enhance patient care protocols. This case contributes to the growing body of evidence supporting vigilant assessment and comprehensive management of CVST in the postpartum population.
